# Identifying health care access barriers in southern rural Ecuador

**DOI:** 10.1186/s12939-022-01660-1

**Published:** 2022-04-22

**Authors:** Anthony Brusnahan, Majo Carrasco-Tenezaca, Benjamin R. Bates, Rosellen Roche, Mario J. Grijalva

**Affiliations:** 1 Infectious and Tropical Disease Institute, Department of Biomedical Sciences, Heritage College of Osteopathic Medicine 204 Grosvenor Hall, Ohio University, Athens, OH 45701 USA; 2 Centro de Investigación Para La Salud en América Latina (CISeAL), Escuela de Ciencias Biológicas, Facultad de Ciencias Exactas Y Naturales, Pontificia Universidad Católica del Ecuador, Quito, Ecuador; 3 School of Communication Studies, Scripps College of Communication, Ohio University, Athens, OH USA; 4 Department of Primary Care, Heritage College of Osteopathic Medicine, Ohio University, Cleveland, OH USA

**Keywords:** Health care access barrier model, Ecuador, Rural population, Underserved population, Health literacy, Financial barriers, Structural barriers, Cognitive barriers, Access to health care

## Abstract

**Background:**

Access to professional health care providers in Loja Province, Ecuador can be difficult for many citizens. The Health Care Access Barrier Model (HCAB) was established to provide a framework for classification, analysis, and reporting of modifiable health care access barriers. This study uses the HCAB Model to identify barriers and themes impacting access to health care access in southern rural Ecuador.

**Methods:**

The research team interviewed 22 participants and completed 15 participant observation studies in the study area. Interviews and a single focus group session of artisans were recorded and transcribed from Spanish to English, and thematic analysis was performed.

**Results:**

The thematic analysis found financial, structural, and cognitive health care access barriers. Cost of medications, transportation, missed responsibilities at work and home, difficulty scheduling appointments, and misconceptions in health literacy were the predominant themes contributing to health care access. These pressure points provide insight on where actions may be taken to alleviate access barriers.

**Conclusion:**

Modifiable health care access barriers outlined in the HCAB are evident in the study area. Further research and implementation of programs to resolve these barriers, such as the creation of health care subcenters and/or mobile clinic, insurance coverage of specialized care, increasing availability and accessibility to affordable transportation, improving roadways, introduction of a 24/7 call center to schedule medical visits, monetary incentive for primary care physicians to practice in rural and underserved areas, provision of affordable work equipment, and emphasizing the improvement of health care literacy through education, may diminish current barriers, identify additional barriers, and improve overall health in the rural area of Loja, Ecuador and similar rural regions around the world.

**Supplementary Information:**

The online version contains supplementary material available at 10.1186/s12939-022-01660-1.

## Background

Health care outcomes are directly related to access to health care [1]. The Health Care Access Barriers (HCAB) Model is one particular method used to address health care access barriers. While there are numerous models designed for this very purpose, the HCAB Model incorporates access barriers as units of analysis, classifies barriers, and provides a framework to facilitate measurement, analysis, and reporting. The HCAB Model identifies three categories of modifiable health care access barriers: financial, structural, and cognitive. In turn, themes can be identified within these categories. This model is not comprehensive, but rather targets modifiable health care access barriers and themes for root-cause analysis and implementation of community-based interventions [2]. By improving overall health, the productivity of societies and economies may also flourish [3]. Additionally, by using a model which focuses on the local level, such as the HCAB Model, we can better understand individual perception and utilization of health care services. In turn, resources and programs to address health care access barriers may be better suited to address local needs [4]. The HCAB Model has been successfully used among minoritized populations in the Global North [1, 2] and in populations in the Global South [3–6]. These studies identify common structural barriers (low educational attainment), financial barriers (cost of service and logistical arrangements) and cognitive barriers (lack of trust and low health literacy) as action points for community-based interventions. In this study, we sought to apply the HCAB Model to underserved communities in rural Loja province, Ecuador.

Generally, access to health care is a concern for rural health populations [3]. Twenty-two percent of Ecuador’s population lives below the income poverty line of $1.90 US dollars (USD) per day [7], and roughly 36% of the population, which is over 6 million people, live in the rural regions of the country [5]. Of those that live in rural areas, 43% live in poverty compared to the 15.9% in urban areas [8]. Poverty is associated with higher unemployment, underemployment, lower salaries, and limited access to markets and productive assets [8]. Furthermore, rural areas are drastically medically underserved, with more than 86% of public practice health care providers and more than 96% of private practice providers working in urban areas [4]. Because of these economic and service-related disparities, health outcomes in rural Ecuador are often worse when compared to urban Ecuador. Examples include: higher rates of preventable injuries, prolonged illness, shorter life expectancy, and generally greater morbidity [7, 9].

The rural population uses three types of professional health care services. The Ministry of Health (MSP) and the Nacional Social Security Institute (IESS) are the larger providers. MSP services are provided free of charge to the entire population, and those enrolled in IESS pay monthly fees of $8–10 USD per month to the “*Seguro Social Campesino.” Seguro Social Campesino* is one of the four main insurance programs in Ecuador. The four programs include: *Seguro de Salud (Health Insurance)*, *Seguro Social Campesino (Social Insurance for Farmers)*, *Seguro de Riesgos de Trabajo (Work Hazard Insurance)*, and *Seguro de Pensiones (Pension Insurance)* [8]. *Seguro Social Campesino* covers physician visits, diagnostic tests, treatment, pregnancy, childbirth and the puerperium, recovery and rehabilitation, disability, retirement pensions, dental care, and assistance with funeral costs [9]. According to the IESS, onefifth of Ecuadorians are covered by one of the four social security schemes [7]. In addition to the services covered by *Seguro Social Campesino*, *Seguro de Salud*, an employer-sponsored health insurance plan, also covers surgical operations and provides monetary subsidies when illness causes an incapacity to work [10].

Tertiary care hospitals are located at the provincial capitals, such as Loja city. Secondary care hospitals are located in the county seats, such as Cariamanga. Health centers with permanent staff are located in larger towns. The health facilities in Calvas County can be seen in Table 1. Itinerant health services are offered at sub-centers located in outlier neighborhoods of cities or in smaller rural communities. The location of these facilities is determined by population size, which leaves people living in smaller communities at a significant disadvantage. Such subcenters include dispensaries of *Seguro Campesino* (Table 2). Private clinics and practitioners are located in cities and operate in a pay per service model. Private providers do not offer service outside of the cities. Informal health care via natural healers and self-medication is common practice in rural areas [11, 12]. The sole hospital accepting only private insurance is Tamayo Day Hospital in Cariamanga, Sucre, and Bernardo Valdivieso (Table 3) [13]. It is no surprise that there are so few health care workers located in the rural area given the aforementioned distribution of the number of rural versus urban health care providers.

**Table 1:** Calvas County Health Facilities

City	Facility	Personnel
Cariamanga	Polyclinic Infantry Brigade Number 20 of the Armed Forces	1 doctor
		1 dentist
		3 nursing assistants
	José Miguel Rosilio Hospital	7 residents
		4 specialty doctors
		9 nurses
		14 nursing assistants
		1 X-ray technologist
		5 laboratory technicians
		1 user/ tech support
	Cariamanga Center of Health	3 general practitioners
		3 general rural practitioners
		1 disability qualifying general practitioner
		1 family doctor
		5 nurses
		3 nursing assistants (2 in dentistry, 1 in pharmacy)
		3 rural dentists
		1 primary health care technician and statistics assistant
Colaisaca	Health Center at Colaisaca	2 general practitioners
		2 nurses
		1 dentist
		2 primary care technicians
		1 nursing assistant
El Lucero	El Lucero Health Subcenter	1 general practitioner
		2 nursing assistants
Sanguillín	Sanguillín Health Post	2 general practitioners
		1 nursing assistant
		1 dentist
		2 nurses
	Usaime Health Post	1 general practitioner
		1 nursing assistant

Source: GAD Municipal Calvas, 2020 and PDOT Parishes/ Cities 2019. Elaboration: Team FEDES, 2020

**Table 2:** Dispensaries of *Seguro Campesino* (*Insurance for Farmers*)

City	Facility	Personnel
Cariamanga	Cariamanga Outpatient Medical Unit	19 general practitioners
		1 laboratory technician
		1 dentist
		1 nurse
		1 nursing assistant
	San Pedro Mártir Communal Medical Dispensary	2 doctors
		1 dentist
		1 nursing assistant
	Yambaca Nongora Farmer’s Social Security Medical Clinic	1 doctor
		1 dentist
		1 nursing assistant
El Lucero	El Tablón Medical Dispensary	1 doctor
		1 dentist
		1 nursing assistant
Utuana	Farmer’s Social Security Dispensary	1 doctor
		1 dentist
		1 nursing assistant

Source: GAD Calvas County, 2020 and PDOT Parishes/ Cities, 2019. Elaboration: Team FEDES, 2020

**Table 3:** Infrastructure of Private Health

Name	Location	Personnel
Tamayo Day Hospital	Cariamanga; Sucre and Bernardo Valdivieso	1 surgeon
		1 pediatrician
		1 general practitioner
		3 traveling doctors (trauma, gynecology, and anesthesiology)
		3 nursing assistants
		1 nurse
		1 laboratory technician

Source: Team FEDES, 2020. Elaboration: Team FEDES, 2020

Although these national-level findings indicate there is a lower availability of health care facilities and that poverty and rurality are likely strong drivers of lower access, each community experiences healthcare delivery differently. Broadly generalized solutions may not address local needs for access. Therefore, we used the HCAB Model to identify themes within the financial, structural, and cognitive barriers to healthcare access faced by community members in three rural communities of Loja, Ecuador.

## Methods

### Study location

**Fig. 1:**
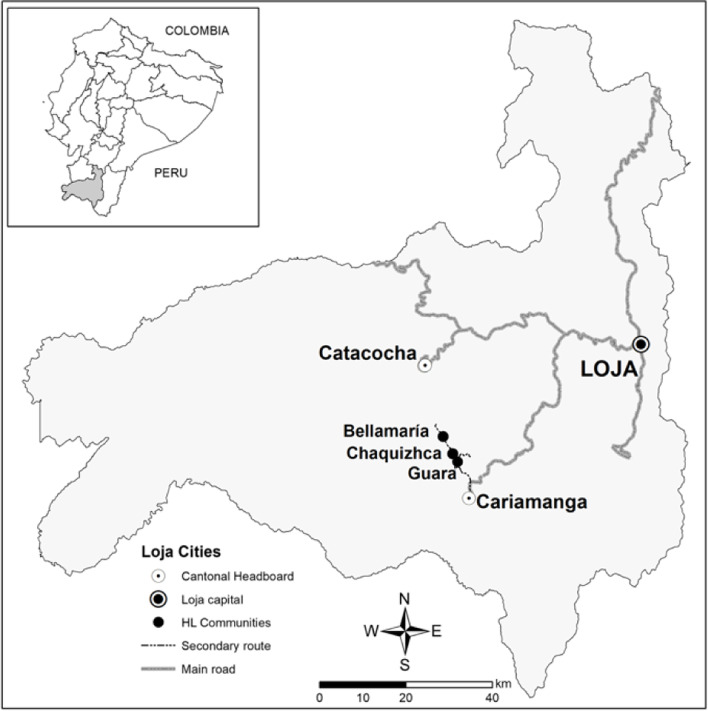
Map of Loja Province, Ecuador. Map highlighting the location of the rural communities of Bellamaría, Chaquizhca, and Guara along with the cities of Cariamanga (Calvas county) and Catacocha (Paltas county) that offer health care facilities, and Loja City (provincial capital) in Loja Province, Ecuador. Created by Cesar A. Yumiseva from CISeAL, PUCE

**Fig. 2:**
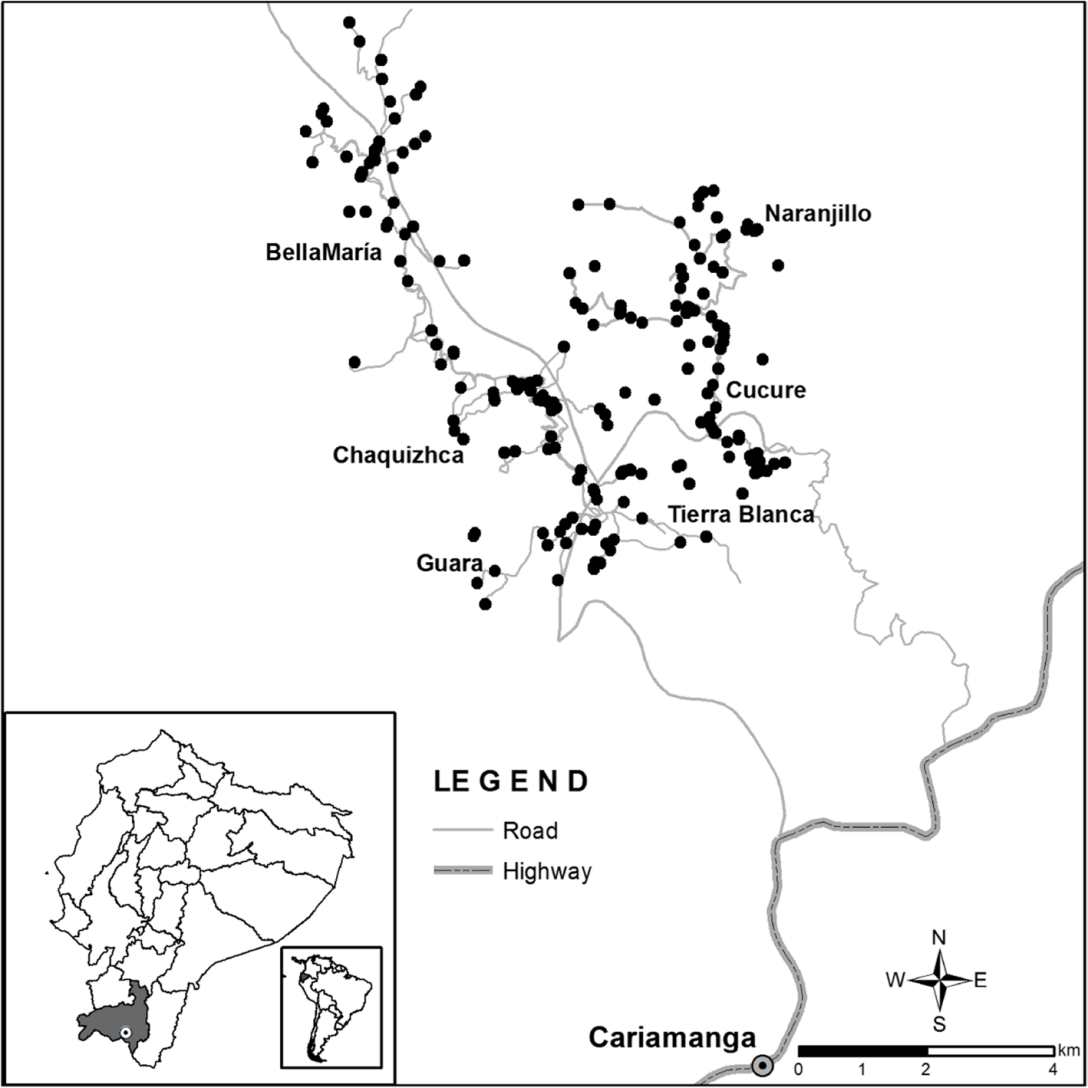
Roads adjacent to the rural communities**.** Map highlighting the roads surrounding the rural communities of Bellamaría, Chaquizhca, and Guara along with the city of Cariamanga (Calvas county). Created by Benjamin Bates from ITDI and Scripps College of Communication at Ohio University

This study was carried out in three rural communities (Bellamaría, Guara, and Chaquizhca), located in Calvas County of Loja Province, Ecuador [6] (Fig. 1) during July of 2017. These three communities are physically and socially isolated due to the deep mountain ridges and poorly maintained dirt roads dividing them. The road network between the communities and Cariamanga can be viewed in Fig. 2. Guara, Chaquizhca, and Bellamaría are located at 9, 14 and 21 kilometers (km) from Cariamanga, respectively. During the dry season, it takes approximately 45, 60 or 90 minutes (min), respectively to traverse these distances by car. During the rainy season, access is hampered by frequent landslides, mud, and general road deterioration. An open window bus, known as “*ranchera”* provides service in and out of the communities once every day. A total of roughly 120 households reside in the three communities, with about 40 in Guara, 35 in Chaquizhca, and 45 in Bellamaría. Members of the community generally practice subsistence agriculture or work as day laborers. Previous research indicates that these communities face limited employment opportunities, poor access to services, and their isolation and marginalization prevent full economic and social engagement with the nearest city, Cariamanga [10, 14]. Cariamanga (pop. ~ 25,000) is itself relatively isolated as Loja, Ecuador, the nearest city of more than 100,000 people, lies roughly 103 km to the northeast. Travel can range from 3 hours (h) by bus or 2 h by car or taxi service. Because of the mountainous terrain and windy roads, it takes most drivers about three hours to make the journey between Cariamanga and Loja.

### Procedures

To understand the financial, structural, and cognitive barriers faced by members of these three communities in accessing health care, two methodologies were employed. A qualitative study, informed by ethnographic approaches and grounded theory, was conducted through a series of semi-structured interviews and participant observations. We used the traditional version of grounded theory where we discovered emerging theories to explain a social process, such as health care access barriers [15]. Additionally, the compatibility of ethnographic studies and grounded theory has been recognized and discussed in literature, This was incorporated in our coding process, which lead to the emergence of themes [16].

Informed consent was obtained before initiating the interviews and/or participant observation studies following a protocol approved by the Institutional Review Board of Ohio University and Pontificia Universidad Católica del Ecuador. These forms were written in Spanish for the participants who were then asked to sign the form. One copy of the form was kept for our record while a second copy was given to the participants.

### Sampling procedures: semi-structured interviews

Seventeen individual semi-structured interviews, as well as a focus group of five artisans, were able to be conducted over the course of four weeks in Cariamanga, and the surrounding rural communities of Bellamaría, Chaquizhca, and Guara. The 22 interviewees were contacted at their homes or places of work regarding their interest in being interviewed and were provided details about the study. The focus group among artisans was conducted at Bellamaría’s community center. The interviews and focus group were conducted at the convenience of the community member. Interviews and the focus group were held at the home or at the place of work of participants and were conducted individually or in small groups. Questions were asked in English and interpreted into Spanish. The subsequent answers from respondents were then interpreted to English. Direct translation from Spanish to English occurred during the transcription of the interviews and focus group from audio recordings. Many of the participants had met our translator from their previous work in the region This made for a more comfortable environment for the participants as the interviewer had no previous relationship with any of the participants. Questions asked during the interview and focus group were concentrated on attaining information regarding respondents’ daily living and occupation to identify potential barriers to health care access. Additionally, direct questions about the HCAB Model’s financial, structural, and cognitive health care access barriers were asked (Supplemental file 1). Interviews and the focus group lasted anywhere from 30 to 90 min given the responses of the participants.

Throughout the interviews, focus groups, and participant observations, we sought to ensure the rigor of our data collection procedures. We obtained saturation by conducting interviews among members of the community until themes began to repeat and no new themes emerged. The credibility of community member response was established via method and analyst triangulation. Multiple forms of data collection allowed for a more complete understanding of the daily lives, and therefore health care access barriers, of the community members. Additionally, the utilization of multiple (two) analysts allowed for verification of the presence of each theme. Auditability, also called confirmability, was assessed by keeping audio-recordings of all interviews and adhering to a semi-structured interview guide. We included the questions to our semi-structured interview in Supplemental File 1 to allow transparency in our study for our readers. Furthermore, we maintained scratch notes of the context and setting of the interviews, focus groups, and participant observations. Finally, transferability was established by thoroughly describing the research context. Our use of the HCAB Model and the description of the setting of these three rural communities creates the baseline for our transferability assumptions.

### Participant observation

Fifteen participant observation sessions were conducted over the course of four weeks at Cariamanga and the surrounding rural communities. Participant observation involved assisting and watching individuals perform their daily tasks. The research team noted the working environment, interactions between workers and their surroundings, relationships with co-workers, tools utilized, and other findings while in the field using descriptive observation [14]. Observed working environments include but are not limited to: plumbing, making panela, working in crop fields, riding the ranchera, and welding. Participant observations were performed to better understand the daily life of respondents and collect more personal, empathetic information that might have otherwise been overlooked during the interviews and focus group. These observations lasted anywhere between 1 to 10 h.

Similar to our interviews and focus groups, we sought to ensure the rigor of our ethnographic procedures. We stopped after fifteen observations as saturation had been attained. The credibility of community member response was established by method and analyst triangulation in addition to cross-analysis with the verbal responses during interviews and focus groups. Auditability was assessed by creating scratch notes during the observations and interviews, confirming them with the translator immediately following the observation, and then writing up more complete field notes each day after observations were completed. Because the translator was known to members of the community, they were able to introduce the first author to members of the community to lessen perspective of intrusion. The second, third, and fifth authors have worked with these communities for several years and are well-known to community members. Finally, transferability was using the same procedures as for the interviews.

### Data analysis

Interviews and observational notes were coded and cross-coded using open coding methods [17]. Following Glaser’s recommendations for grounded theory analysis, the HCAB Model was used as a sensitizing framework [18, 19]. Using the concept of “barriers” as the primary sensitizing concept and the HCAB Model’s identification of financial, structural, and cognitive barriers as starting points, participant talk and observational data were sorted into coding families under these three barriers. This approach allows for theoretically-informed categorization but does not force data into these categories, as additional barriers not anticipated in the HCAM were allowed to emerge. Coding was performed by two independent reviewers. One coder conducted the interviews and focus group, and the other coder was not associated with data collection but was familiar with the work. If a new theme appeared to emerge for a coder they discussed it with the other coder until consensus that a new theme had emerged or that it fit within one of the barriers identified by the HCAB Model. In addition, although the three barriers informed code families, they did not determine every member of those families. Coders, in sorting through participant talk, placed statements into a code family and, if there were discrepancies between coders, they discussed the categorization until consensus was reached. Coding was performed using ATLAS.ti Version 8.4.4 Mac.

### Data management

Transcriptions were analyzed using ATLAS.ti Version 8.4.4 Mac (ATLAS.ti Scientific Software Development GmbH) and then stored in a secure database.

## Results

### Population background

**Fig. 3:**
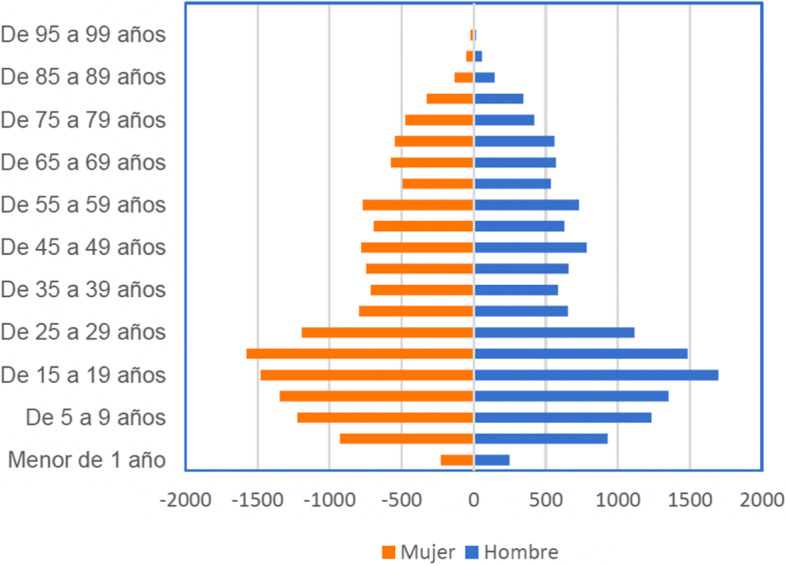
Projection of the population by sex and age of Calvas county to 2020. Source: INEC 2010; Team FEDES, 2020. Elaboration: Team FEDES, 2020. Translations (Spanish: English). de: from; años: years; mujer: woman; hombre: man

The estimated population of Calvas County in 2020 based on age and sex can be viewed in Fig. 3. Of note, most of the population lies within the age range of 15–30 years old. The majority of the respondents in our study were male (*n* = 13) and were involved in animal farming (*n* = 14) or agriculture (*n* = 14). Agriculture was divided into self-employed or employed by an agriculture company. Those who were self-employed worked on their one own plot or offered their services locally. Male respondents were more likely to work in agriculture (*n* = 11) than women (*n* = 3), but both men and women were similarly involved in animal farming (*n* = 8 and *n* = 6, respectively). Agriculture and animal farming provided a source of income from sales at the local market in Cariamanga as well as sustenance. Livestock kept by the local citizens included animals for labor and travel. Female respondents were more likely to work as artisans (*n* = 5) and/or perform household chores (*n* = 9), especially when compared to men (*n* = 0 and *n* = 3, respectively). Most participants reported having multiple occupations, which is why Table 4 does not add up to a total of 22 participants. Furthermore, Table 4 did not include a separate sex/gender column as the majority of males worked in agriculture, and the majority of females had occupations based indoors such as teacher, maid, and artisan. Additional occupations as well as the percentage of each occupation stated among those included in the semi-structured interviews, the focus group, and participant observation can be seen in Table 4.

**Table 4:** Self-reported occupation of local citizens of three rural communities in Loja Province, Ecuador (*n* = 22, total; *n* = 13, male; *n* = 9, female)

Variable	Male n (%)	Female n (%)	Total n (%)
***Type of work***			
Agriculture (self-employed)	10 (77%)	3 (33%)	13 (59%)
Animal Farming	8 (62%)	6 (67%)	14 (64%)
Agriculture (for company)	1 (8%)	-	1 (5%)
Mechanic	1 (8%)	-	1 (5%)
Fishing	1 (8%)	-	1 (5%)
Artisan	-	5 (56%)	5 (23%)
Water system	3 (23%)	-	3 (14%)
Driver	1 (8%)	-	1 (5%)
Teacher	-	1 (11%)	1 (5%)
Maid	-	1 (11%)	1 (5%)
Household chores	3 (23%)	9 (100%)	12 (55%)

### Overview of HCAB

Through thematic analysis of direct quotations in interviews, the focus group, and experiences during participant observations, themes were identified among financial, structural, and cognitive health care access barriers based on the Health Care Access Barriers Model [2] (Table 5) using an inductive approach based in grounded theory.

**Table 5:** Themes across Health Care Access Barriers (*n* = 22, total; *n* = 13, male; *n* = 9, female)

Variable	Male n (%)	Female n (%)	Total n (%)
***Financial Barriers***			
Lack of health insurance	2 (15%)	1 (11%)	3 (14%)
Cost of transportation	4 (31%)	2 (22%)	6 (27%)
Cost of medications	2 (15%)	2 (22%)	4 (18%)
Cost of internet to schedule appointment	-	1 (11%)	1 (5%)
Cost of telephone call to schedule appointment	1 (8%)	1 (11%)	2 (9%)
Needs to financially support family	10 (77%)	4 (44%)	14 (64%)
***Structural Barriers***			
Travel time requires missing work	10 (77%)	8 (89%)	18 (82%)
No one to do house chores	2 (15%)	8 (89%)	10 (45%)
No one to take care of animals	9 (69%)	6 (67%)	15 (68%)
Distance/ travel time for appointment	4 (31%)	2 (22%)	6 (27%)
Lack of child/ elderly care	4 (31%)	5 (56%)	9 (41%)
Wait time at the clinic/ hospital	3 (23%)	4 (44%)	7 (32%)
Multiple locations for tests and specialists	3 (23%)	1 (11%)	4 (18%)
Difficulty scheduling an appointment	5 (38%)	5 (56%)	10 (45%)
Few ambulances	1 (8%)	-	1 (5%)
***Cognitive Barriers***			
Low health literacy	13 (100%)	8 (89%)	21 (95%)
Lack of preventative measures	10 (77%)	3 (33%)	13 (59%)
Elects to ignore health care advice	4 (31%)	1 (11%)	5 (23%)
Practices presenteeism	13 (100%)	9 (100%)	22 (100%)
Self-medicate and/or go to healer	13 (100%)	9 (100%)	22 (100%)
Unsatisfied with care	3 (23%)	4 (44%)	7 (32%)

### Financial barriers

Themes for financial barriers included but were not limited to the following: need to support family financially, cost of transportation, and cost of medications. The need to support one’s family was the main deterring financial barrier in seeking health care (64%), with a distinct difference between men (77%) and women (44%). For one individual alone, the minimum cost for an entire trip via *ranchera* could end up being 4% or more of one’s monthly salary. Even when traveling by other means, the cost of gas for driving or riding a personal vehicle per day was equivalent to a round-trip to Cariamanga via *ranchera*. The cheapest way of getting to Cariamanga was by walking or riding a donkey, assuming the donkey was already purchased.

Lastly, four interviewees (18%) also stated having difficulty affording medications with some medications reportedly costing thousands of dollars. One interviewee stated her father’s surgery would have cost $3000 USD instead of $110 USD if the doctor didn’t change the paperwork from an appointment in their clinic to a consultation from the emergency department. Another individual receiving chemotherapy reported some of their medications cost up to $2000 USD, and their specialist appointments were $60-$70 USD each. Even during an emergency, one individual without insurance chose to take their donkey from Cariamanga to pick up an injured member in the communities instead of calling a truck due to cost. However, most of the respondents (86%) had health insurance, and 82% of individuals went to physicians and/ or hospitals that accepted *Seguro de Salud* or *Seguro Social Campesino* health insurance. Health insurance companies cover the majority of costs at clinics that accept their plans but will not always cover the entirety of cost at private clinics [7]. Of the three uninsured respondents, one had enough money to go to a private clinic while the other two were finalizing paperwork for health insurance. Of note, both interviewees who saw private physicians stated it was easier to get an appointment and patient wait time was significantly lower when compared to clinics and hospitals for patients with *Seguro de Salud* or *Seguro Social Campesino* insurance (Table 6).

**Table 6:** Locals with health insurance and type of professional care provided (*n* = 22)

Variable	Total n (%)
***Have health insurance***	22
Yes	19 (86%)
No	3 (14%)
***Professional health care provider***	22
Physician/ hospital accepting *Seguro de Salud* or *Seguro Social Campesino*	18 (82%)
Private physician	2 (9%)
Emergency room	3 (14%)
Occupation’s health care center	1 (5%)

### Structural barriers

Themes for structural barriers included but were not limited to: inability to care for family members, address household chores, or tend to animals, lack of available appointments with healthcare providers, distance to clinics and hospitals, limited number of seats on the ranchera, and lack of number of ambulances. As represented in Table 5, time spent away from home to seek health care caused 82% of respondents to miss work (77% of men and 89% of women), 68% were unable to take care of animals, 45% couldn’t perform household chores, and 41% could not care for their children or elderly parents. Even when patients had access to health care providers, nearly half of respondents (45%) had difficulty scheduling an available appointment.

One respondent stated, “When we’re very, very sick, we make an appointment for 2 or 3 days later. Making an appointment is very difficult. The appointment book is usually full. You need to have a real emergency to make an appointment with short notice.” Additionally, another interviewee mentioned, “You go, and you might not get the appointment that day so I’ll come back [*home*] without appointment or without seeing the doctor."

Those who go to facilities accepting health insurance can sometimes acquire an appointment for the following day at the earliest. One interviewee reported that a clinic didn’t have appointments available for six days, and they were forced to go to the hospital in Cariamanga instead. To further note the difficulty in scheduling an appointment, clinics were not open on the weekends and many physicians at the hospital had weekends off. Even when patients had appointments at clinics that accepted health insurance, they sometimes waited two to four hours before being seen at the *Seguro Social Campesino* clinic in Chiriguala. Only two (9%) interviewees had regularly scheduled appointments with professional health care and one of these respondents had additional regularly scheduled appointments for chemotherapy. A review of Tables 1, 2 and 3 notes the limited number of health care providers in Calvas County, which contributes to the limited number of appointments that can be scheduled by members of the community.

Six people went to the *Seguro Social Campesino* clinic in Chiriguala and four people went to specialists in cities other than their primary care location. One individual used a motorbike to get to a doctor in Catacocha (130 km away). Visits to specialists required a notable amount of time, effort, and money.

For example, one respondent stated, “I get to Loja by bus, and then I stay in my daughter’s house. She then calls for a taxi, and the taxi takes me to SOLCA [Tertiary cancer care hospital]. That is not that near [*to get to the hospital*].”

The *ranchera* was utilized by twenty-one of the twenty-two interviewees (95%). The only individual who did not take the *ranchera* lived in Cariamanga and did not have family residing in the rural communities. The *ranchera* was very limited in the number of available seats. With only 25 seats across three stops, the individuals in Chaquizhca and Guara, the last two stops, had a more difficult time getting a seat on the *ranchera*. Most respondents (55%) stated they used the bus from Cariamanga for travel to other cities. Half (6) of the people who used the bus went to the *Seguro Social Campesino* clinic in Chiriguala (15 km away from Cariamanga).

Despite having an emergency ambulance service, which consisted of three ambulances covering Cariamanga and the surrounding communities, none of the interviewees had used it. The ambulances belong to the government; one is specifically linked to *Seguro Social*, and the other two run on behalf of the Ministry of Public Health. If available, the ambulance service is provided at no cost. Additionally, these vehicles are used to transport patients to Loja City. However, the ambulances were often unavailable as they frequently required repairs. An interviewee expressed their concern over the service stating “they [ECU 911 operators] sometimes say that the (three) ambulances are in another place so they cannot come.” In this particular circumstance, the respondent had to hire a truck during a gout flare because an ambulance was not available. The other modes of transportation, destinations, reasons for travel, and cost can be viewed in Table 7.

**Table 7:** Mode of transportation (*n* = 22)

Variable	Total n (%)	Destination	Reason for Travel	Cost
***Mode of transportation***				
Personal vehicle	4 (18%)	Cariamanga Cantón Balao Catacocha	Work in Cariamanga (3) Work in Cantón Balao (1) Health care in Catacocha (1)	$3.00 per day for gasoline^a^
Truck for hire	4 (18%)	Cariamanga	Work (3) Health care (1)	$10-$25
*Ranchera*	21 (95%)	Cariamanga	Health care (8) Work (6) Health care at another location (10) Visit family (3)	$0.75 from Guara $1.00 from Chaquizhca $1.75 from Bellamaría $1.00 average price for bag $0.25 for young child $0.50 for older child
Walking	6 (27%)	Cariamanga Within the communities	Work in Cariamanga (4) Work in the communities (5)	Free
Taxi ruta	2 (9%)	Loja	Health care in Loja (1)	$10-$11 from Cariamanga to Loja
		Cuenca	Health care in Cuenca (1)	$12 from Loja to Cuenca
Bus	12 (55%)	Loja Quito Riobamba Cariamanga Chiriguala	Health care in Chiriguala (6) Visit family in Cariamanga (1) Buy machinery and attend workshops in Riobamba (1) Health care in Loja (2) Pass through Loja to health care in Cuenca (1) Woodwork workshop in Loja (1) Electrician workshop in Quito (1)	$4.00 from Cariamanga to Loja $10.50 from Cariamanga to Guayaquil
Donkey	3 (14%)	Cariamanga Within the communities	Emergency room at Cariamanga (2) Work in the communities (1)	Free (excluding purchase of donkey)

^a^estimated cost to drive or ride on any given day given gasoline prices per Rhue (2016)

Number in parenthesis expresses number of respondents who stated that reason for travel

Cantón Balao: a city in the province of Guayas, Ecuador

### Cognitive barriers

The most common themes identified as cognitive barriers to health care access were: low level of health literacy, use of traditional medications and self-treatment, and lack of preventative measures. Perception of health can influence understanding of when to utilize health care resources, resulting in a cognitive barrier to health care access. 95% of interviewees did not have an education in the health care field, which may have contributed to a lower level of health literacy. One respondent explained the immune system was not taught in the primary school located in the communities. As a result, some of the locals do not believe in the immune system. One interviewee stated locals believe, “diseases are consequences of the polluted air because of insecticide spreading or because it is god's will.” Another individual mentioned how “the illnesses or virus are getting more resistant, so natural medicine… doesn’t work.” She believes this is why more people in the communities have started going to health care professionals rather than solely relying on traditional remedies. However, she also believed “if you walk on the Earth without shoes, you release the bad energies” and you “get more defenses, like the immune system.”

When asked, “What do you believe are the most common medical conditions in the community?” there were a wide variety of answers. Most interviewees agreed upon influenza (57%) with a similar response between men (54%) and women (63%). Sixty-two percent of male respondents mentioned unhealthy trends in diet. Similarly, 50% of female respondents believed the use of chemicals in the production of food caused illness among community members. Besides diet, most male respondents also associated illness with exposure to the sun (54%) with a noticeable dichotomy compared to female respondents (25%) (Table 8).

**Table 8:** Perception of common medical conditions and why individuals get sick (*n* = 21, total; *n* = 13, male; *n* = 8, female)

Variable	Male n (%)	Female n (%)	Total n (%)
***Most common medical conditions in the community***^a^	13	8	21
Influenza	7 (54%)	5 (63%)	12 (57%)
Common cold	7 (54%)	3 (38%)	10 (48%)
Back Pain	5 (38%)	2 (25%)	7 (33%)
Cancer	2 (15%)	1 (13%)	3 (14%)
Diabetes mellitus	3 (23%)	-	3 (14%)
Fever	3 (23%)	-	3 (14%)
Gastritis	1 (8%)	2 (25%)	3 (14%)
Headache	3 (23%)	-	3 (14%)
Arthritis	1 (8%)	1 (13%)	2 (10%)
Hypercholesterolemia	2 (15%)	-	2 (10%)
Hypertension	2 (15%)	-	2 (10%)
Malaria	2 (15%)	-	2 (10%)
Diarrhea	-	1 (13%)	1 (5%)
Myocardial infarction	1 (8%)	-	1 (5%)
Stroke	1 (8%)	-	1 (5%)
***Why individuals get sick***	13	8	21
Unhealthy diet/ Lack of nutrients/ Eating too much	8 (62%)	2 (25%)	10 (48%)
Exposure to the sun	7 (54%)	2 (25%)	9 (43%)
Eating chemically treated food	4 (31%)	4 (50%)	8 (38%)
Don’t care for their health/ go for periodic check-ups	3 (23%)	1 (13%)	4 (19%)
Spreading insecticide	4 (31%)	-	4 (19%)
Being stressed	1 (8%)	1 (13%)	2 (10%)
Exposure to dust	2 (15%)	-	2 (10%)
Not drinking enough water	2 (15%)	-	2 (10%)
Working too much	1 (8%)	1 (13%)	2 (10%)
Alcoholism	1 (8%)	-	1 (5%)
Antimicrobial resistance	-	1 (13%)	1 (5%)
Being from the city	1 (8%)	-	1 (5%)
Born sick	1 (8%)	-	1 (5%)
Changes in weather	1 (8%)	-	1 (5%)
Destiny	1 (8%)	-	1 (5%)
Low immune system	1 (8%)	-	1 (5%)
Walk on the dirt without shoes	-	1 (13%)	1 (5%)

^a^Self-reported medical conditions during the interview and focus group

When identifying common medical conditions in the communities, 57% of respondents stated influenza and 48% mentioned the common cold (Table 9). Additionally, back pain (33%) and headaches (14%) were attributed to the physical demand of one’s job and occupational exposure, respectively. An estimated 23% of interviewees elected to ignore health care advice. Some stated they didn’t need the treatment or used traditional medicine instead. Every interviewed member self-medicated with either traditional medicine, saw a healer, used OTC medications, or a combination of the three. "They [the people of Loja] usually self-medicate, so they buy medicines in the pharmacy to treat the flu." Sometimes the reasoning for these self-medicated treatments were inaccurate or even harmful. Many of the traditional medicine remedies are based on “hot” or “cold” theories, which were remedies tested in a trial-and-error fashion and passed down through generations.

**Table 9:** Medical conditions of local citizens and their treatment regiments

**Variable**	**Treatment Options**^a^
***Medical Condition***	
Cut hand	Anti-inflammatory pill, OTC
	Chamomile tea (externally applied)
Cut leg	Chamomile tea (externally applied)
	Rubbing alcohol (externally applied)
	Pain medication, prescribed
	Tetanus injection
	Hospital-administered medication to prevent infection
Influenza	Orange juice, hot
	Lemonade
	Quemado
	Alcohol (ingested)
	Guichingue plant seed, flower of Tilo plant, lemon juice, and honey in boiling water (ingested)
	Sangorache (amaranth flower) juice or tea with lemon
	Tea from Llantén plant
	Mortiño berries (blue berries)
	Singripal pill, OTC
	Coffee with liquor
Gastritis	Chamomile tea (ingested)
	Buscapina (ingested)
	Aspirin
	OTC medications
	Alcohol (ingested)
	Achotillo fruit in boiled water
Diabetes mellitus	Aspirin
	Modifying diet
Hypertension	Anti-hypertensive pills (undefined)
Hypercholesterolemia	Prescribed medications (undefined)
Hernia	Surgery
Dengue	Prescribed medications (undefined)
	Yerbatera healer injections (undefined)
Kidney stone/ dehydration	Surgery
	Hydration
	Prescribed medications (undefined)
	Myroxylon balsamum/ Chaquino plant stem in boiled water, allowed to cool
Body/ back pain	Stop heavy lifting
	Prescribed medications (undefined)
	Herbal tea (undefined)
	Swimming
	Alcohol (ingested)
	Shower
	Therapy exercises
	Anti-inflammatory pills, OTC and prescribed
	Rest
	Curandera healer
	Menthol
Headache	Prescribed medication
	Finalín pill, OTC
Common cold	OTC medications
	Lemonade, hot
	Tea from Llantén plant
	Sugar cane
	Mortiño berries
	Buscapina pill with oregano
	Moshquera with oregano
	Quemado
	Sangorache juice or tea with lemon
Gout	Cucumbers
	Avoid red meat
	Medication, in hospital
	Prescribed medication
	OTC medication
Side effects of spreading insecticide	Warm shower
	Rest
	Eat a meal
	Sangorache juice or tea
Thyroid cancer	Thyroidectomy followed by levothyroxine and chemotherapy
Appendicitis	Appendectomy
Fever	OTC medication
	Sangorache juice or tea with lemon
	Tea from Llantén plant
	Mortiño berries
Pneumonia	Prescribed amoxicillin and paracetamol

^a^*Definitions:*

*OTC*: over-the-counter

*Horchata:* herbal tea drink made from a mix of herbs and flowers. Herbs include but are not limited to cola de caballo/ shave grass, llantén, borraja, flax, and escancel/ bloodleaf. Flowers include but are not limited to rose geranium, small roses, violets, begonias, carnations, fuschias, and malva olorosa/ malva blanca. [20]

*Lemonade*: horchata with lemon

*Quemado*: hot distilled alcohol/ guarapo (fermented sugar cane juice), panela (unrefined whole cane sugar), orange juice; some add cinnamon or lemon

Guichingue: plant; species is *Bidens pilosa* [21]

*Tilo*: herb; species is *Justicia pectoralis* [21]

*Sangorache*: plant; species is *Amaranthus cruentus L.* [21]

Llantén: plant; species is *Plantago major L.* [21]

Mortiño berries: fruit; species is *Solanum americanum Mill.* [21]

*Singripal*: acetaminophen, pseudoephedrine hydrochloride, chlorpheniramine maleate, dextromethorphan hydrobromide, ascorbic acid

*Achotillo*: fruit; species is *Caryocar amygdaliferum Mutis* [21]

*Yerbatera/Curandera*: healer who uses plant remedies

*Buscapina*: acetaminophen, caffeine, pyrilamine maleate

*Finalín*: caffeine and acetaminophen

*Moshquera*: bitter herb; species is *Croton sp.* [22]

*Chaquino*: plant; species is *Myroxylon balsamum *[23]

A number of respondents (59%) either did not know or did not practice one or multiple preventative measures based on interviews, the focus group, and participant observation. When asked what makes them feel good, an overwhelming 73% said working; 56% said being and/or working with fellow members of the community; and 14% said doing well at work. Only 18% stated not being ill. All respondents practiced presenteeism, or going to work while ill or injured, and had elected to self-medicate and/or go to a healer before seeking western medical council at some point in their life. The following quotation is a prime example of the multi-factorial reasoning behind why many people in rural Loja do not see a physician on a frequent basis:

“Sometimes because we work so much that we don't take care of our health or the children’s well-being. We don't visit the doctor regularly; we just go when we feel really bad and that's because we need to work to provide for our families.”

Table 9 displays the medical conditions experienced by interviewees and the treatment regiments they utilized.

## Discussion

As previously stated, improving overall health can help the productivity of societies and economies. Additionally, by using the HCAB Model, we can better understand individual perception and utilization of health care services which may result in the development of resources and programs that directly address local needs.

### Financial barriers

With most community members working in agriculture and/ or animal farming, it is difficult for them to get away from their occupational responsibilities without also losing money, food, and supplies that come from their labor. One’s personal health becomes a tradeoff between placing food on the table, providing for their family, and caring for their crops and animals. Additionally, many community members sell their goods at the local market in Cariamanga, which requires payment for transportation of themselves and their items to be sold. So, for many community members to make a profit from their goods, they need to pay for transportation to and from the city. On an average income of $40 USD per month, time away from work to spend money on travel, appointments, and medications can be detrimental to a household’s financial state. Given this information, it is no surprise that 100% of interviewees practiced presenteeism.

One solution to this financial barrier is government-sponsored transportation. This would offer members of the community transportation at reduced or no cost. One such program would be similar to the Transportation Reimbursement Incentive Program in Riverside County, California who has provided 1.6 million free trips to the elderly and disabled. This program offers incentive for friends and neighbors to use their own vehicles to transport others [24]. Although the practice of hiring neighbors for transportation has been practiced in the communities, this program would allow free transportation to those who might otherwise not have an immediate method of travel. However, given that there are not enough vehicles in the community to support such a program, additional forms of transportation would need to be offered.

The cost of care also impacted accessibility to health care. With some surgeries costing $3000 USD, individual chemotherapy treatments at $2000 USD, and specialist appointments of $60-$70, it is no wonder that the majority of community members cannot afford proper health care, and as a result, foregoes treatment. A proposed recommendation would be a health insurance plan that covers the majority of cost for specialized care.

One study in the rural county of La Maná, Ecuador displayed how the creation of a health subcenter significantly reduced non-medical expenses, such as travel cost, and diminished the financial barrier for health care access [25]. A health subcenter was previously suggested in the communities but was declined [7]. This is because although these communities are rural and of difficult access due to road conditions, they do not qualify to have a subcenter because are located close enough to the City of Cariamanga, where a hospital is located. Even if a health subcenter could not be implemented in the communities, a local pharmacy or mobile clinic, such as the Health Wagon [26], has the potential to limit health care access barriers by reducing financial burden and travel for care.

### Structural barriers

Structural barriers revolved around time spent away from home. It takes 45–90 min to travel Cariamanga, which offers the nearest hospital, secondary schools, local government, administrative centers, and market for crops and livestock. The *ranchera*, leaves the communities at 5 AM and begins its return back at 2 PM. This meant most people were away from home for at least nine hours. In addition to missing work, many did not have anyone to watch their children, elderly parents, or animals while they seek health care because they are gone too long. Furthermore, if an individual missed the *ranchera* or if the *ranchera* was full, they were forced to postpone their trip or find another mode of transportation, the latter being much more expensive. Specialized care was further away from the communities, costing residents more of their time. Therefore, individuals would practice presenteeism, take traditional and OTC medications accessible within their own homes, and go to local healers before traveling to a healthcare professional in the city.

Studies performed in Ecuador and the United States have recognized transportation as a health care access barrier [7, 27, 28] and have suggested introducing or increasing public transit to combat this barrier [27]. Joint action with non-health sectors, such as the *ranchera* company, will likely be necessary to alleviate this structural barrier [29]. For example, the number of *rancheras* could be increased, allowing for more residents to travel at once as well as during different times of the day.

Since the introduction of the *Ministerio de Salud Publica’s* policy of requiring health care appointments to be scheduled online or via telephone, it has been increasingly difficult for community members to get an appointment. As a result, community members are unable to be seen in a timely fashion for their basic health needs. This decreases work productivity, which can lead to a lower number of crops sold and less food to feed their families. Furthermore, they can get other family members sick if their illness is communicable. To make scheduling appointments easier, a 24/7 call center may be established. Community members who did not have telephones were able to use a neighbor’s phone without issue. Although more medical providers would need to offer services in Cariamanga in order for more appointments to be available, at least community members would easily be able to schedule a medical visit. A program like the Ohio Physician Loan Repayment Program could be established to increase the number of primary care physicians in the area. Such a program would have the government provide monetary incentive for primary care providers to work in rural and underserved areas such as Cariamanga [30].

Lastly, there was a significant issue with the availability of ambulances in the community. The service consisted of only three vehicles covering a vast area: Cariamanga, the communities in the study, and many other communities around Cariamanga. During emergent situations, the citizens of Bellamaría, Chaquizhca, and Guara had to hire trucks or ask their neighbors for a ride. Considering that emergent care was 45–90 min away, it is increasingly difficult to get those in critical conditions to a facility which can properly care for them. Provided this information, increasing the number of ambulances and improving road conditions would help increase both availability and response times in the communities surrounding Cariamanga.

### Cognitive barriers

Low health literacy has been identified in literature as a prominent health care access barrier for low-income families [31, 32] and was evident in our study through low level of health education, use of traditional medications that could lead to harm in one’s health, and not using preventative measures either due to lack of knowledge, disregard for safety, or inadequate access to safety measures.

As previously stated, 95% of interviewees did not have an education in the health care field, which may have contributed to a lower level of health literacy. Furthermore, the immune system was not taught in the primary school located in the communities. Therefore, it is not surprising that most reasons as to why people got sick centered around working too much, poor diet, and occupational exposure to the sun, insecticide, and dust rather than to microorganisms and the immune system. Yet, agricultural activity which exposes workers to dust and insecticide can cause respiratory disorders [33], and literature has demonstrated a positive relationship between agricultural work and pulmonary disorders [34]. Additionally, while an unhealthy diet can cause complications such as heart disease, diabetes, nutrient deficiency, and obesity, it does not directly cause infection or the spread of communicable diseases. Yet, both can lead to a state of reduced immune response, leading to increased susceptibility to infection. However, even with accurate diagnoses and explanations from professional health care providers, almost a quarter of the interviewees elected to ignore health care advice or utilized traditional medicine. Families used “hot” and “cold” medications to “equilibrate” the temperature of their illness. On a few occasions, traditional or OTC medication would lead to a harmful effect, such as alcohol and aspirin utilized as treatment for gastritis, or an was an ineffective treatment, like aspirin used to treat diabetes mellitus. Everyone self-medicated in some fashion. This could lead to the prolongation and worsening of one’s illness. However, if an adult’s condition persisted or worsened or if their children were ill they would attempt to see a health care provider in the clinic or hospital.

Lack of preventative measures noted during participant observation contributed to some of the medical conditions in the community. On numerous occasions, locals knew they were putting themselves at risk but either elected not to take appropriate precaution or could not afford the equipment to do so. Some examples include: a welder not using a face shield or gloves, a fisherman walking in the river at night without light, workers of the water system using a saw without a handle, and people drinking alcohol while at work.

According to Brazil’s National Commission on Social Determinants of Health, a higher schooling level influences the use of health services, access to treatments, understanding of health and information problems, and adhesion to a healthier lifestyle [35]. Additionally, numerous studies have shown how education at the community level can play a pivotal role in improving health care access and decreasing unfavorable health outcomes. A handful of teaching lessons on health, hygiene, first aid, healthy lifestyle factors, and preventative measures was suggested by a study in Romania that incorporated the HCAB Model [31]. Educational videos [36], lectures [37], distributing information to take home, and protective equipment packages [38] were found to reduce and prevent certain illnesses and poor health outcomes as well. In particular to the communities in the study, it would be imperative to incorporate the pros and cons to traditional and OTC medications. According to Rhue [7], some programs were developed to provide medical visits consisting of vaccines, dentistry, perinatal care, and more in the communities with the intention of occurring once a week. However, the hospital did not have enough staff, so the visits only happen once or twice a year. Therefore, resources need to be allocated accordingly if programs are to be initiated in the communities.

Although education can be used to teach those who do not understand or knowingly did not take preventative precautions, community members still need to be able to afford the proper equipment. The government could offer a kickback to businesses willing to provide equipment at a reduced cost to rural and underserved areas like the communities around Caraiamanga. The tools used in many of the occupations in the community, including agriculture, tending to animals, and welding pose a high risk for injury, and care for these injuries lies 45–90 min away in the city. Furthermore, injury at work would either lead to lack of or significantly reduced income or sustenance for families. So, preventative safety measures with proper equipment may lead to improved productivity, reduced injuries, and increased ability to provide for one’s family.

### Limitations

Information between interviewees and the interviewers needed to be translated between English and Spanish. Any information lost, as well as any miscommunication, could potentially influence the results of the study. Additionally, this study was based on self-reporting by interviewees. Information that was misconstrued or omitted by the interviewee could impact our results. To help prevent researcher bias, a pre-determined list of questions was created prior to the conduction of the study. Descriptive observation was also utilized to prevent fixation on patterns emerging from the data or pre-conceived prior to the study. Furthermore, the greater percentage of male participants in the study limits the ability to generalize our results. However, the fact that many of the respondents repeatedly stated similar barriers to health care access greatly supports our findings. Additional studies in rural regions both within and outside of Ecuador are important for verification purposes.

## Conclusion

This study uses the Health Care Access Barriers Model to show that there are identifiable themes within financial, structural, and cognitive barriers that limit health care access in rural communities of Ecuador. The combination of interviews, the focus group, and participant observation helped provide a clear understanding of daily life for residents in the study area. The need to support one’s family, the cost of transportation, and the cost of medication were the predominant financial themes. The most noticeable theme among structural barriers was not having someone available to watch over children, elderly parents, and/ or animals while seeking care for one’s health. Additional themes in structural barriers were limited availability of seating on the ranchera, inability to get appointments with healthcare providers, long distances to healthcare providers, and lack of ambulances. Lastly, the primary themes among cognitive barriers were low health literacy, use of traditional medications and self-treatment, lack of preventative measures taken.

From this study, it is evident that there are prevalent themes in financial, structural, and cognitive health care access barriers which exist in Cariamanga and the surrounding rural communities in Loja Province, Ecuador. The HCAB Model and variations of the model have successfully been utilized in a number of previous studies. As a result, programs could be implemented to directly address the now recognized barriers. Examples include but are not limited to: establishing a health subcenter and/or mobile clinic, increasing public transit or different methods of public transportation, developing insurance plan which would reduce cost of specialized care, creating a 24/7 call center to make medical appointments, making a government-sponsored monetary incentive program to increase primary care physicians in rural and underserved areas, offering affordable equipment for improved productivity and safety measures, encouragement and incentives for locals to obtain higher level of education, implementing health classes at the community level, providing information such as pamphlets for citizens of the community to read, and health care packages for locals to take home. Through the utilization of the HCAB Model, we hope additional research will be conducted in the study area and similar regions around the world. Researchers can review the description of our research methods as well as the background of our research context to help recreate the study. In turn, further health care access barriers may be identified so that community-level interventions, including those suggested in our manuscript, may be implemented to mitigate them. As a result, overall health outcomes can be improved in Ecuador’s Loja Province and comparable rural areas around the world.

## Supplementary Information

**Additional file 1:** **Supplemental file 1.** Semi-structured Interview Questions.

## Data Availability

The data analyzed during the study is not publicly available as it contains private information regarding the interviewees and individuals from participant observation studies. The data is available upon personal request with the exclusion of said private information.

## Abbreviations

HCAB Model: Health Care Access Barrier Model
OTC: Over-The-Counter

## References

[1] Cohen J (2003). Disparities in health care: an overview. Acad Emerg Med.

[2] Carrillo JE, Carrillo VA, Perez HR (2011). Defining and targeting health care access barriers. J Health Care Poor Underserved.

[3] Strasser R (2003). Rural health around the world: challenges and solutions*. Fam Pract.

[4] López-Cevallos, D. F., & Chi, C. Assessing the context of health care utilization in Ecuador: a spatial and multilevel analysis. BMC Health Serv Res. 2010;10(1). 10.1186/1472-6963-10-64 10.1186/1472-6963-10-64 PMC2850335 20222988

[5] Index Mundi. 2018. Ecuador - Rural population. Retrieved March 27, 2020, from https://www.indexmundi.com/facts/ecuador/rural-population

[6] Nieto-Sanchez C, Baus EG, Guerrero D (2015). Positive deviance study to inform a Chagas disease control program in southern Ecuador. Mem Inst Oswaldo Cruz.

[7] Rhue, S. 2016. The Effects of Distance on Community Health and Chagas Disease. (Electronic Thesis or Dissertation). Retrieved from https://etd.ohiolink.edu/

[8] Instituto Ecuatoriano de Seguridad Social. 2019. Retrieved 13 Apr 2020, from https://www.iess.gob.ec

[9] Instituto Ecuatoriano de Seguridad Social. 2019. Seguro Campesino. Retrieved 13 Apr 2020, from https://www.iess.gob.ec/es/seguro-campesino

[10] Instituto Ecuatoriano de Seguridad Social. 2019. Seguro de Salud. Retrieved 13 Apr 2020, from https://www.iess.gob.ec/es/18

[11] Aldulaimi S, Mora FE (2017). A primary care system to improve health care efficiency: lessons from Ecuador. J Am Board Fam Med.

[12] Granda ML, Jimenez WG. The evolution of Socioeconomic Health Inequalities in Ecuador during a Public Health System Reform (2006–2014). International Journal for Equity in Health. 2019;18(1). 10.1186/s12939-018-0905-y 10.1186/s12939-018-0905-y PMC6368770 30736808

[13] FEDES. Plan de Desarrollo y Ordenamiento Territorial del canton Calvas 2019–2023. PDYOT 2019–2023. https://www.gobiernocalvas.gob.ec/index.php/sistema-informacion-local/territorial/pdyot-2019-2023. Published 1 June 2021. Accessed 23 Jan 2022.

[14] Worldometer. 2020. Ecuador Population (2020). Retrieved 27 Mar 2020, from https://www.worldometers.info/world-population/ecuador-population/

[15] Annells M (1996). Grounded theory method: philosophical perspectives, paradigm of inquiry, and postmodernism. Qual Health Res.

[16] Pettigrew SF. Ethnography and Grounded Theory: a Happy Marriage? Advances in Consumer Research. 2000;27:256–60. Retrieved January 23, 2022, from https://www.acrwebsite.org/volumes/8400/volumes/v27/NA-.

[17] Saldaña J (2011). Fundamentals of qualitative research.

[18] Glaser Barney (1978). Theoretical Sensitivity Advances in the Methodology of Grounded Theory.

[19] Glaser Barney (1992). Emergence vs. Forcing: Basics of Grounded Theory Analysis.

[20] Pujol L, says DGM, Markham DG, et al. Horchata Lojana: Ecuadorian herbal tea drink. Laylita's Recipes. https://www.laylita.com/recipes/horchata-lojana/. Published January, 2020. Accessed 13 Apr 2020.

[21] Padilla-Herrera Andrea (2020). Knowledge, usage and potential commercialization of medicinal plants in Calvas county in the province of Loja in Ecuador.

[22] Aguirre Z. Especies forestales de los bosques secos del Ecuador. . COIN - Food and Agriculture Organization of the United Nations. https://coin.fao.org/coin-static/cms/media/21/14042335632720/especies_forestales_bosques_secos_del_ecuador.pdf. Published March 2012. Accessed 14 Apr 2020.

[23] Bailon-Moscoso N, Romero-Benavides JC, Tinitana-Imaicela F (2015). Medicinal plants of Ecuador: a review of plants with anticancer potential and their chemical composition. Med Chem Res.

[24] USA.gov. 2019. Rural Public Transportation Systems. U.S. Department of Transportation. Retrieved January 23, 2022, from https://www.transportation.gov/mission/health/ Rural-Public-Transportation-Systems

[25] Gaus DP, Herrera DF, Mantyh WG (2011). Quantifying the reduction in nonmedical costs after the introduction of a rural county hospital in Ecuador. Rev Panam Salud Pública.

[26] Gardner T, Gavaza P, Meade P, et al. Delivering free healthcare to rural Central Appalachia population: the case of the Health Wagon. Rural and Remote Health. 2012;12:2035. Retrieved from https://www.rrh.org.au/journal/article/2035 22452285

[27] Del Rio M, Hargrove W, Tomaka J (2017). Transportation matters: a health impact assessment in rural New Mexico. Int J Environ Res Public Health.

[28] Syed ST, Gerber BS, Sharp LK (2013). Traveling towards disease: transportation barriers to health care access. J Community Health.

[29] HirmasAdauy M, Poffald Angulo L, JasmenSepúlveda AM (2013). Health care access barriers and facilitators: a qualitative systematic review. Rev Panam Salud Pública.

[30] Ohio Department of Health. (2022). Rural Health Information Hub. Funding Details: Ohio Physician Loan Repayment Program. Retrieved January 23, 2022, from https://www.ruralhealthinfo.org/funding/3090

[31] George S, Daniels K, Fioratou E. A qualitative study into the perceived barriers of accessing healthcare among a vulnerable population involved with a community centre in Romania. Int J Equity Health. 2018;17(1). 10.1186/s12939-018-0753-9 10.1186/s12939-018-0753-9 PMC5883264 29615036

[32] Lazar M, Davenport L (2018). Barriers to health care access for low income families: a review of literature. J Community Health Nurs.

[33] Zejda JE, McDuffie HH, Dosman JA, Epidemiology of health and safety risks in agriculture and related industries (1993). Practical applications for rural physicians. West J Med.

[34] Linaker C (2002). Respiratory illness in agricultural workers. Occup Med.

[35] National Commission on the Social Determinants of Health. 2008. The social causes of health inequities in Brazil. Retrieved March 27, 2020, from http://bvsmig5.icict.fiocruz.br/wp-content/uploads/2016/02/sum_exec_en.pdf

[36] Abiodun OA, Olu-Abiodun OO, Sotunsa JO, et al. Impact of health education intervention on knowledge and perception of cervical cancer and cervical screening uptake among adult women in rural communities in Nigeria. BMC Public Health. 2014;14(1). 10.1186/1471-2458-14-814 10.1186/1471-2458-14-814 PMC4133628 25103189

[37] Wang JM, Xiong WN, Xie JG (2016). Impact of village-based health education of tobacco control on the current smoking rate in Chinese rural areas. J Huazhong Univ Sci Technolog Med Sci.

[38] Laoraksawong P, Sanpool O, Rodpai R, et al. Impact of the health education and preventive equipment package (HEPEP) on prevention of Strongyloides stercoralis infection among rural communities in Northeast Thailand: a cluster randomized controlled trial. BMC Public Health. 2018;18(1). doi: 10.1186/s12889-018-6081-6 10.1186/s12889-018-6081-6 PMC6194667 30340481

