# Supplementary figures and images for: Identifying health care access barriers in southern rural Ecuador
Source: Int J Equity Health. 2022 Apr 22;21:55. doi: 10.1186/s12939-022-01660-1 (PMC9027412; doi:10.1186/s12939-022-01660-1)

**Supplemental file 1. Semi-structured Interview Questions**


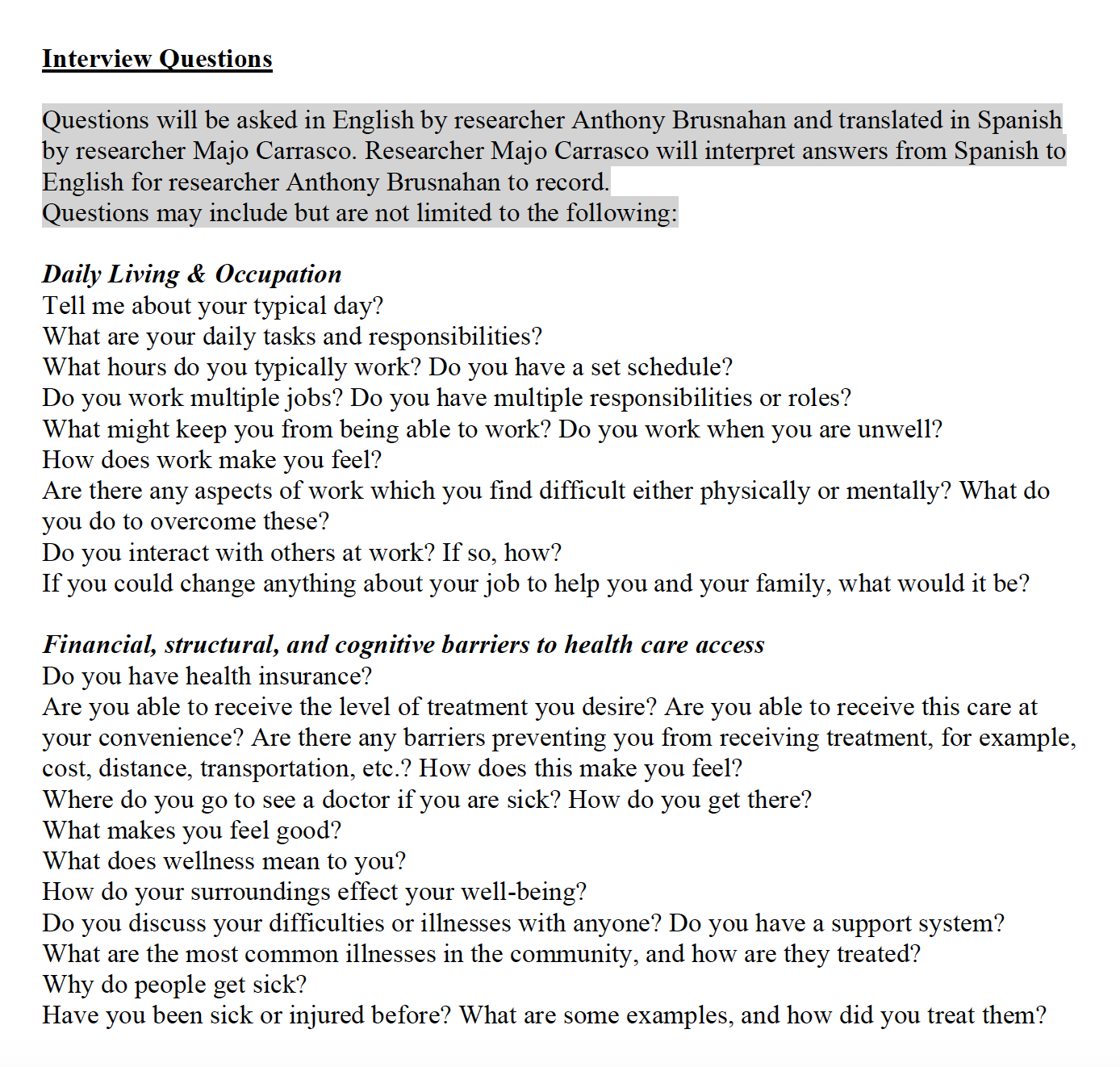

Supplement: Supplementary file 1 — Additional file 1: Supplemental file 1. Semi-structured Interview Questions. [file 12939_2022_1660_MOESM1_ESM.docx]
